# Supplementary material for: Pellucidin A promotes antinociceptive activity by peripheral mechanisms inhibiting COX-2 and NOS: In vivo and in silico study
Source: PLoS One. 2020 Sep 17;15(9):e0238834. doi: 10.1371/journal.pone.0238834 (PMC7498071; doi:10.1371/journal.pone.0238834)
Supplement: Attachment — Submitted filename: Response letter July.docx [file pone.0238834.s002.docx]

**Response Letter**

**Reviewer #1:** While the authors have addressed some of the comments, there remain a few important corrections that need to be made:
1. I still don't see any mention of which pain model was used in the mice in the hot plate test. Are these mice uninjured? formalin-treated? please specify.

**Response:**

The hot plate test has been widely used as a measure of supraspinal antinoception in several studies (Lamberts, 2013, Pasternak, 2001). The thermal or chemical agents just induce pain without injure mice . The time between the placement of the mouse on the platform and shaking, licking of the paws or jumping was recorded as the hot-plate latency, to clarify in Hot-plate test (2.9), Materials and Methods, line 200, you can read “Licking or jumping is a rapid response to painful thermal stimuli that is a direct indicator of nociceptive threshold”, page 09. Also, was explain in formalin test.

2. Page 14, line 304. it still states "insert p value here"

**Response:**

We corrected the sentence according to the reviewer’s suggestion. The sentence was changed and values were insert

3. The Discussion is extremely long and confusing. It would benefit from reorganization for clarity. For example, iNOS is mentioned in multiple different paragraphs that are not linked. Please go through and make sure there is a logical flow to the Discussion in order to highlight only the most salient points of the paper.

**Response:**

We corrected the discussion according to the reviewer’s suggestion.

**Reviewer #2:** The authors have addressed all the concerns raised in the original submission. There is no further comments.

**Response:**

Thanks
